# Thermal stability and anisotropic thermal expansion of WS_2_ annealed in different atmospheres

**DOI:** 10.1039/d6ra02325h

**Published:** 2026-07-15

**Authors:** Doyeon Jin, Peiting Wen, Yi Li, Ulrich Kentsch, Oliver Steuer, Shengqiang Zhou, Slawomir Prucnal

**Affiliations:** a Institute of Ion Beam Physics and Materials Research, Helmholtz-Zentrum Dresden-Rossendorf 01328 Dresden Germany jindoyeon459@gmail.com s.prucnal@hzdr.de

## Abstract

Two-dimensional transition metal dichalcogenides (TMDCs) are promising semiconductors for next-generation nanoelectronic and optoelectronic devices due to their thickness-dependent band structure and direct band gap in the monolayer limit. However, their integration into CMOS-compatible platforms requires exposure to high-temperature processing under various ambient conditions, raising concerns about their thermal stability and structural robustness. Here, we systematically investigate the effects of high-temperature annealing on the structural and optical properties of monolayer WS_2_. Temperature-dependent photoluminescence and micro-Raman spectroscopy reveal a strong dependence of lattice dynamics, thermal expansion, and degradation pathways on the annealing atmosphere. Samples annealed in air exhibit triangular etch pit formation at approximately 650 K, accompanied by pronounced anisotropic thermal expansion. In contrast, WS_2_ annealed under argon shows nearly isotropic thermal expansion and remains structurally stable up to ∼950 K. We demonstrate that degradation initiates at sulphur-deficient regions, such as flake edges and intrinsic sulphur vacancies, which act as nucleation centres for oxidative decomposition. The triangular pits align along metal-terminated zigzag directions, indicating anisotropic bond dissociation governed by defect chemistry and edge energetics. These results provide critical insight into atmosphere-dependent thermal degradation mechanisms and establish guidelines for the reliable integration of TMDCs into high-temperature semiconductor processing.

## Abstract 2

Air induces anisotropic expansion and rapid oxidative etching, while inert environments preserve structural and optical integrity. Gemini was used when creating the GA image.

## Graphical abstract

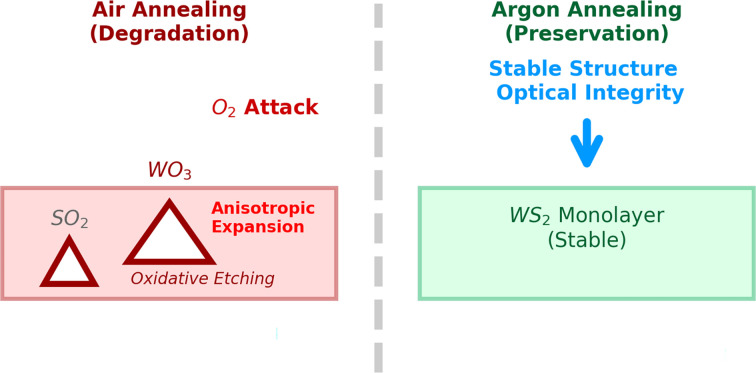

## 1. Introduction

The family of two-dimensional (2D) materials comprises several thousand systems with band gaps ranging from the deep ultraviolet (*e.g.*, hexagonal boron nitride, hBN) to zero band gap materials, such as graphene. Among them, transition metal dichalcogenides (TMDCs) constitute a prominent class of atomically thin semiconductors with the general formula MX_2_, where M is a transition metal (*e.g.*, Mo, W) and X is a chalcogen (S, Se, or Te). Most unintentionally doped TMDCs exhibit n-type conductivity.^1^ This behaviour is primarily attributed to extrinsic factors, including cross-contamination, surface states, and the adsorption of oxygen or other environmental species.^1–3^ Although chalcogen vacancies are commonly present, they typically act as deep donor states that trap charge carriers rather than effectively contributing free electrons.^4^ The electrical conductivity of 2D materials can be tailored through both *in situ* and *ex situ* doping strategies. *In situ* approaches involve the incorporation of foreign elements during material growth, whereas *ex situ* methods are applied post-synthesis.^5–9^ Among *ex situ* techniques, thermal diffusion and intercalation are most commonly employed.^10^ For example, Yi Li *et al.* demonstrated p-type doping of monolayer WS_2_*via* phosphorus ion implantation followed by flash lamp annealing,^11^ while chlorine ion implantation has been used to enhance n-type doping in MoSe_2_.^12^ Das *et al.* reported efficient p-type doping of WSe_2_ through chemisorption of NO molecules at selenium vacancy sites.^13^ Considerable attention has also been devoted to the thermal behaviour of monolayer and few-layer TMDCs under vacuum and oxygen atmospheres.^14–18^ Notably, two-layer WS_2_ grown by chemical vapor deposition has been shown to withstand annealing temperatures up to at least 775 °C.^19^ With increasing annealing temperature, a reduction in sample resistivity and an improvement in crystallinity have been reported. In contrast, Lei Wang *et al.* annealed monolayer MoS_2_ in an argon-diluted sulphur atmosphere and observed a continuous enhancement of photoluminescence intensity up to 850 °C, with the threshold degradation temperature (the temperature at which the structural degradation of the flake was observed) estimated at approximately 860 °C.^20^ Annealing above this threshold leads to the formation of triangular defects aligned along Mo-terminated zigzag (Mo-ZZ) edges, which are energetically more favourable.^21^ The size of these triangular defects increases progressively with both annealing temperature and time. Xuewen Wang *et al.* systematically investigated the influence of substrate choice, Al_2_O_3_, SiO_2_, and mica, on the thermal stability of mechanically transferred MoS_2_.^22^ Annealing in air at atmospheric pressure resulted in triangular pit formation at temperatures as low as 290 °C. In vacuum, pit formation in few-layer samples occurred at approximately 350 °C, while monolayer flakes exhibited slightly enhanced stability, with initial pit formation observed near 300 °C. Annealing in air under reduced pressure further improved thermal stability up to 350 °C. Substrate-dependent behaviour was also evident: samples transferred on mica showed reduced thermal stability, whereas MoS_2_ transferred on Al_2_O_3_ was the most stable. The rapid degradation of TMDCs in oxygen-containing environments is primarily attributed to oxidation–reduction reactions between O_2_ molecules and the TMDC lattice.^23^ A comprehensive understanding of the thermal stability of TMDCs and their interaction with ambient environments at elevated temperatures is therefore critical for the reliable development of multifunctional devices.

In this work, we present a systematic study of the thermal stability of CVD-grown WS_2_ on SiO_2_/Si substrates, annealed in air and argon atmospheres. *In situ* micro-Raman spectroscopy reveals that thermal expansion is anisotropic for monolayer and few-layer WS_2_ when annealed in air, whereas it is nearly isotropic under argon. The thermal stability of WS_2_ is strongly dependent on the annealing environment: samples annealed in argon remain stable up to 950 K (∼670 °C), while annealing in air leads to complete etching of the flakes at 650 K (∼380 °C).

To further investigate the role of defects, we studied a set of ion-irradiated samples. The results show that thermal stability decreases significantly with increasing ion fluence, and etching initiates at the flake edges even during ion irradiation. These observations indicate that point defects, such as vacancies and dangling bonds, critically determine the thermal stability of thin TMDC layers. Based on these findings, we conclude that the fabrication of defect-free TMDCs and the protection of flake edges, for example *via* capping layers such as h-BN or more common dielectric like high-k materials *e.g.* Al_2_O_3_, are essential for achieving thermally stable and reliable devices.^11^ This work highlights the combined influence of annealing atmosphere and defect density on the structural integrity of TMDCs, providing practical guidance for their integration into high-temperature semiconductor processes.

## 2. Experimental part

Two types of WS_2_ flakes were used in this study: CVD-grown flakes on SiO_2_/Si substrates and mechanically exfoliated flakes. Flake thickness was initially determined from the frequency difference between the two main Raman modes, E^1^_2g_ (in-plane) and A_1g_ (out-of-plane), and subsequently confirmed by atomic force microscopy (AFM). Mechanically exfoliated flakes were specifically used for ion-induced defect experiments. In this case, WS_2_ flakes were mechanically transferred onto 90 nm thick SiO_2_ on Si substrates, grown by dry thermal oxidation, using the conventional scotch-tape method. Following transfer, samples were sequentially cleaned in acetone, isopropanol, and deionized water, and finally dried under nitrogen. Exfoliated WS_2_ flakes were implanted with phosphorus ions at fluences of 5 × 10^12^ cm^−2^ and 1 × 10^13^ cm^−2^ with an ion energy of 7 keV. To confine the implanted P ions within the monolayer WS_2_, a 7 nm thick Al_2_O_3_ capping layer was deposited by atomic layer deposition (see ref. 11 for details). After implantation, the Al_2_O_3_ capping layer was removed by etching in 1% HF for 10 seconds, and samples were subsequently subjected to high-temperature annealing in air.

**Fig. 1:**
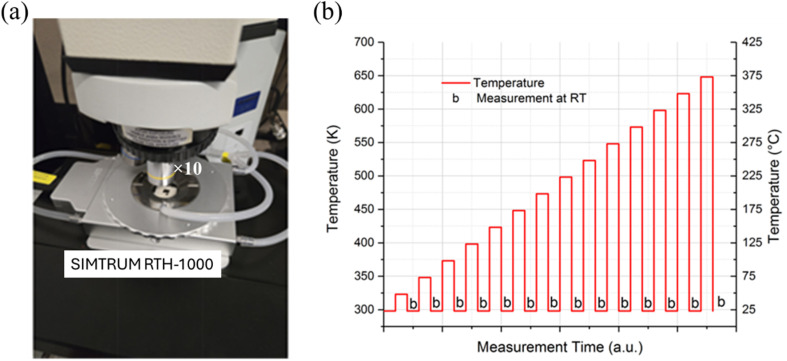
(a) RTH-1000 chamber with sample installed on the optical table of the micro-Raman system and (b) the schematic representation of the heating/cooling process used for the determination of the threshold degradation temperature for TMDCs. In the case of photoluminescence studies, after each high temperature step, sample was cooled to the RT and new recorded PL spectrum was named with “b”.

Thermal treatments for all samples were carried out using a High-Temperature Heating Stage (RTH-1000, SIMTRUM PTE. Ltd, Singapore shown on Fig. 1a). The RTH-1000 offers adjustable heating rate from 0.1 to 30 °C min^−1^, with a temperature stability of ±0.1 °C over the range from room temperature to 1000 °C. Thermal stability of the flakes was evaluated either in air or under a continuous flow of high-purity argon (99.999%) with a flowing rate of 2 sccm (standard cubic centimetres per minute). The threshold degradation temperature was determined *in situ* from changes in the photoluminescence (PL) spectra and the Raman phonon modes, without removing the samples from the annealing chamber. Micro-Raman spectra were recorded with a temperature step of 25 K, starting from room temperature. At each temperature, the sample was stabilized for 5 min before measurement.

Samples were excited using a 532 nm laser, and the scattered signal was collected through a ×10 objective and detected with a liquid-nitrogen-cooled Si-CCD. PL spectra were collected using the same setup, both at elevated temperature and after cooling the samples to room temperature following each annealing step (Fig. 1b). This procedure enabled monitoring of both reversible and permanent changes in optical and structural properties during thermal cycling.

The heating rate was set to 30 °C min^−1^. At each target temperature, the sample was held for 5 min to ensure thermal stabilization and to allow refocusing of the optical system, if necessary, due to thermal expansion of the heating stage. Raman spectra were collected using an acquisition time of 10 s with 10 accumulations. Consequently, the sample remained at each temperature for approximately 7 min, followed by about 1 min required to reach the next temperature step. Therefore, each annealing step lasted approximately 8 min. The same temperature protocol was applied during the photoluminescence measurements. For PL experiments, after each annealing step the sample was cooled to room temperature before the spectra were recorded. Since the cooling time depends on the maximum annealing temperature, the cumulative time spent above room temperature varied between successive temperature steps.

The structural modification of the flakes was monitored with optical microscope Zeiss (Axio Scope.A1) and scanning electron microscope.

## 3. Results and discussion

### 3.1. Micro-Raman investigation

**Fig. 2:**
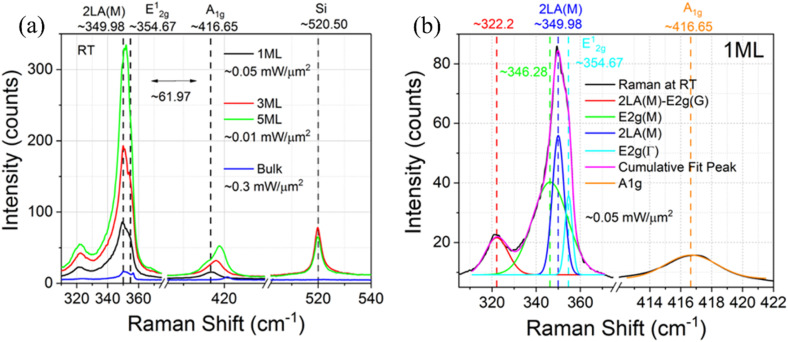
(a) Micro-Raman spectra of WS_2_ with different thicknesses (1 ML, 3 ML and 5 ML describing flakes with different number of layers) and (b) Gaussian deconvolution of the Raman spectrum of monolayer thick (1 ML) WS_2_.

Fig. 2 presents the room-temperature micro-Raman spectra of WS_2_ flakes with varying thicknesses: monolayer (1 ML), trilayer (3 ML), five layers (5 ML), and bulk. For monolayer WS_2_, the two dominant phonon modes, the in-plane E^1^_2g_ mode and the out-of-plane A_1g_ mode, are observed at approximately 354 cm^−1^ and 416 cm^−1^, respectively.^24^ The peak at 520 cm^−1^ originates from the transverse optical phonon mode of the underlying silicon substrate and serves as an internal ref. 25. Detection of the Si phonon is facilitated by the semi-transparent nature of monolayer and few-layer WS_2_ in the visible spectral range and is absent in the spectrum of bulk WS_2_. The number of layers in a WS_2_ flake can be determined from the separation, Δ*ω*, between the E^1^_2g_ and A_1g_ Raman modes. For monolayer WS_2_, Δ*ω* is 61.97 ± 0.18 cm^−1^, while trilayer (3 ML), five-layer (5 ML), and bulk WS_2_ exhibit Δ*ω* values of 62.37 ± 0.13, 63.49 ± 0.30, and 64.35 ± 0.06 cm^−1^, respectively. Gaussian deconvolution of the Raman spectrum for 1 ML WS_2_ (Fig. 2b) reveals additional peaks at approximately 322, 346, 350, and 354.67 cm^−1^. The peaks at 350 and 354.67 cm^−1^ correspond to the 2LA(M) and E^1^_2g_ phonon modes, respectively, while the lower-frequency peaks are commonly attributed to disorder-induced phonon modes.^26^ The 2LA(M) mode arises from the second-order longitudinal acoustic phonon at the M point of the Brillouin zone.^27^

**Fig. 3:**
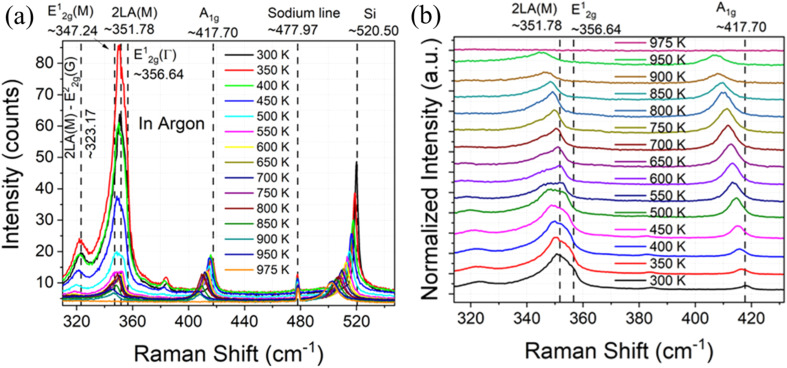
(a) Temperature-dependent micro-Raman spectra of monolayer WS_2_ measured in an argon atmosphere. Dashed black lines indicate the room-temperature peak positions. (b) Normalized Raman spectra highlighting the evolution of the intensity ratio between the 2LA(M) and E^1^_2g_ modes as a function of temperature.

For monolayer WS_2_, the out-of-plane A_1g_ mode appears as a single peak at 416.65 cm^−1^, whereas in few-layer samples, the A_1g_ mode exhibits splitting due to interlayer interactions.^28^Fig. 3a shows temperature-dependent micro-Raman spectra of monolayer WS_2_ annealed in an argon atmosphere. Spectra were recorded in 25 K steps, but the figure presents every second spectrum for clarity. As expected, all phonon modes redshift and their intensities decrease with increasing temperature. A small peak at ∼478 cm^−1^ corresponds to a sodium line from the laboratory lighting, used as a reference. Initially, the Raman intensity increases, reaching a maximum around 350 K, and returns to room-temperature intensity at 400 K. At higher temperatures, intensities gradually decrease, and above 950 K, WS_2_ phonon modes are no longer detectable. The remaining peaks at 975 K correspond to the sodium line (∼478 cm^−1^) and the Si transverse optical (TO) mode (∼520 cm^−1^). Additionally, the intensity ratio between the 2LA(M) and E^1^_2g_ modes evolves with temperature. At room temperature, the 2LA(M) mode dominates, but by ∼550 K, both modes have comparable intensity. At higher temperatures, the ratio inverts, with the E^1^_2g_ mode becoming dominant until complete flake degradation occurs at 975 K (Fig. 3b).

**Fig. 4:**
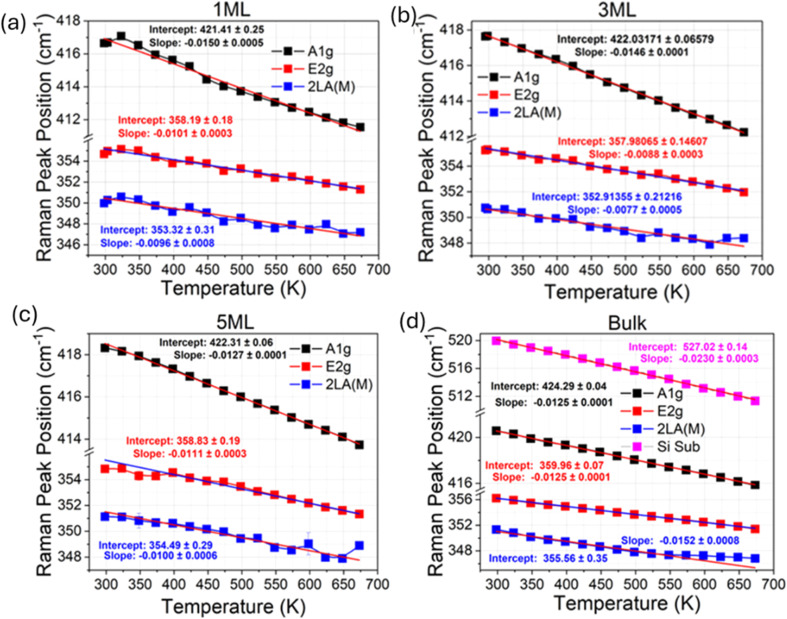
Temperature-dependent shifts of the main Raman phonon modes (E^1^_2g_, 2LA(M), and A_1g_) for WS_2_ flakes with thicknesses of (a) 1 ML, (b) 3 ML, (c) 5 ML, and (d) bulk, annealed in air over the temperature range 300–675 K. The TO phonon mode of the underlying Si substrate is shown in (d) for reference. The intercept corresponds to *ω*_0_, while the slope is proportional to the in-plane or out-of-plane thermal expansion coefficient of the WS_2_ films.

Fig. 4 shows the evolution of the main Raman phonon modes (E^1^_2g_, 2LA(M) and A_1g_) as a function of annealing temperature for WS_2_ flakes of different thicknesses, annealed in air. The data were fitted using a linear function *ω*(*T*) = *ω*_0_ + *χT*, where *ω*_0_ is the phonon frequency at 0 K, *χ* is the first-order temperature coefficient, and *T* is the temperature.^33^ Two key features are observed for flakes annealed in air: (i) the thermal expansion depends on the number of layers, with distinct slopes for the in-plane E^1^_2g_ and out-of-plane A_1g_ modes as a function of WS_2_ thickness, and (ii) clear anisotropic thermal expansion for thin flakes, while bulk WS_2_ exhibits nearly isotropic behaviour. The difference between the slopes of the E^1^_2g_ and A_1g_ modes decreases with increasing layer number and vanishes in the bulk limit. The 2LA(M) mode exhibits a slightly different slope, but its temperature dependence is largely linear; the small deviation at higher temperatures (≥575 K) may arise from the thermal evolution of the deconvoluted components.

Temperature-dependent data for monolayer and bulk WS_2_ annealed in air are provided in Fig. S5. Overall, the 2LA(M) mode remains a sensitive indicator of layer thickness and is influenced by doping, strain, and temperature.^27,34^ Although all flakes are bonded to the Si substrate, the much larger thermal expansion of Si compared to the in-plane expansion of WS_2_ thin films suggests weak interfacial bonding between the flakes and the substrate.

Next, we have calculated the thermal expansion coefficient *α* for WS_2_ with different thicknesses. The *α* parameter of monolayer thick WS_2_ is strongly affected by the substrate while the value obtained for few layers thick WS_2_ should be comparable to the bulk crystal. In principle the in-plane thermal expansion coefficient can be extracted from the E^1^_2g_ slope presented in Fig. 4. We have to take into account that the change of the phonon frequencies *ω* as a function of temperature *T*, 
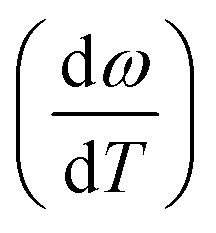
, is due to lattice expansion 
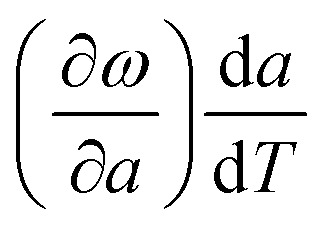
, where *a* is the lattice parameter, and anharmonic effect 
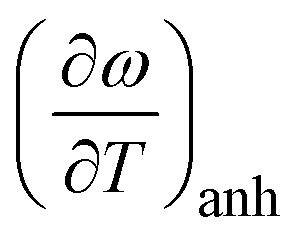
. The thermal expansion coefficient can be expressed as 
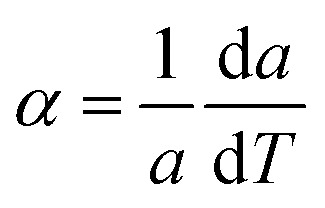
. First, we have to separate the anharmonic and thermal expansion contributions from the slope and next we can calculate *α* according to 
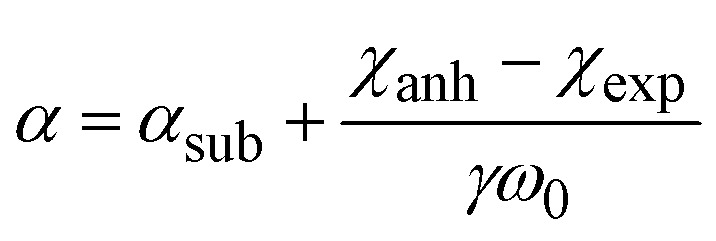
, where *α*_sub_ = 0.5 × 10^−6^ K^−1^, anharmonic slope for in-plane mode E^1^_2g_, *χ*_anh_, is taken −0.007 cm^−1^ K^−1^. The Grüneisen parameter *γ* is assumed to be 0.9176 for E^1^_2g_.^35–37^ The values of *χ*_exp_ and frequency *ω*_0_ for both phonons are indicated in Fig. 4. The obtained *α* parameter for in-plane thermal expansion of monolayer, a few layers thick and bulk WS_2_ are summarised in Table 1.

**Table 1:** In-plane thermal expansion coefficient *α* estimated from temperature dependent Raman spectroscopy

	In-plane phonon mode, E^1^_2g_		
Thickness	*χ*_exp_ cm^−1^ K^−1^	*ω*_0_ cm^−1^	*α* × 10^−6^ K^−1^
1 ML	−0.0101	358.19	7.1 ± 0.2
3 ML	−0.0088	357.98	6.2 ± 0.2
5 ML	−0.0111	358.83	7.8 ± 0.2
Bulk	−0.0125	359.96	8.8 ± 0.2

The apparent thermal expansion coefficient increases with thickness, indicating a transition from substrate-dominated strain response in monolayers to intrinsic lattice expansion behaviour in bulk WS_2_. The determination of out-of-plane thermal expansion coefficient for van der Waals crystals is much more complicated since the A_1g_ phonon mode is sensitive to interlayer coupling. The out-of-plane thermal expansion coefficient was estimated from DFT calculations reported by Wilczyński *et al.*, yielding *α*_d_ ≈ 2 × 10^−6^ K^−1^ for the monolayer thickness and *α*_c_ ≈ 2 × 10^−5^ K^−1^ for the interlayer separation.^37^

Assuming that approximately 50% of the phonon softening originates from quasiharmonic thermal expansion, as suggested by DFT calculations, an effective out-of-plane thermal expansion coefficient for a few layers thick WS_2_ is in the range of 3.4–4.1 × 10^−5^ K^−1^. For monolayer the out-of-plane thermal expansion refers to the change of the S–S bonding distance since the monolayer thick van der Waals crystals have no real out-of-plane lattice parameter.

**Fig. 5:**
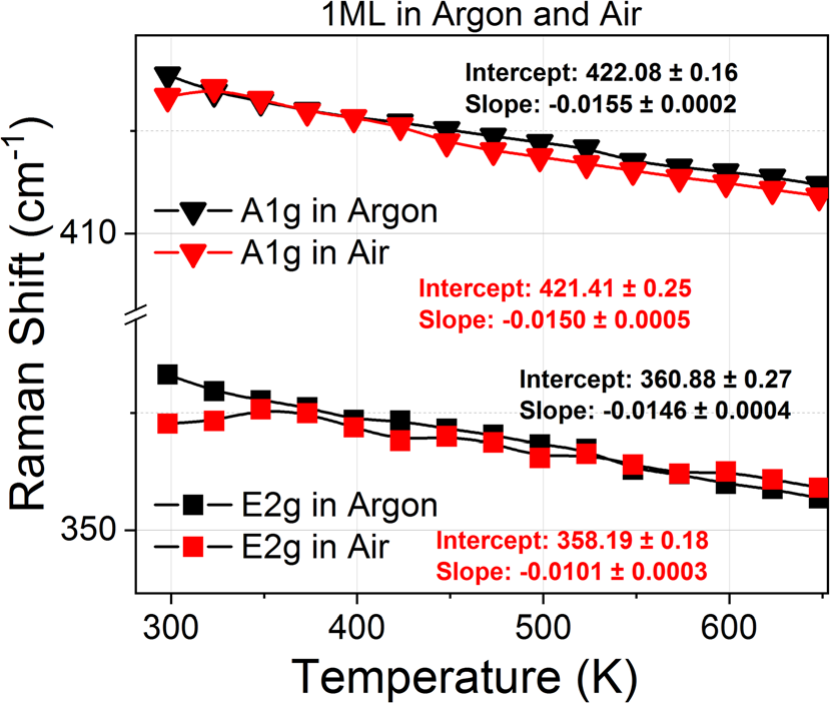
The change of the peak of in-plane (square symbols) and out-of-plane (triangles) phonon modes in monolayer thick WS_2_ as a function of temperature during annealing in either argon (black symbols) or air (red symbols).

Fig. 5 compares the thermal expansion of monolayer WS_2_ annealed in air and in argon. Notably, the slopes of the in-plane (E^1^_2g_), and out-of-plane (A_1g_) phonon modes are nearly identical for the sample annealed in argon, whereas they differ significantly for the sample annealed in air. We attribute this behaviour to chemical interactions between oxygen and the WS_2_ surface. At elevated temperatures, oxygen can first adsorb and subsequently chemically react with monolayer WS_2_, modifying the vibrational modes. The modification of the surface due to chemical interaction of oxygen with the flake is only one of the possible explanations. Due to adsorption of oxygen at the WS_2_ surface the donation of carriers into monolayer flakes takes place. The change of the carrier concentration can be another reason for observed anisotropy due to electron–phonon coupling.^38^ In contrast, argon, as an inert noble gas, does not react with WS_2_ and has a negligible effect on the flake's thermal expansion.

**Fig. 6:**
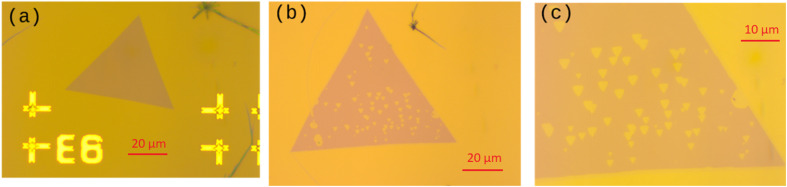
Optical images of as-grown WS_2_ monolayer flake (a) and after annealing in air at 400 °C (b). (c) Shows the magnified image of the flake presented in (b).

Fig. 6 shows optical images of a WS_2_ flake before and after annealing at 400 °C in air. The as-grown flake exhibits the typical triangular morphology and appears defect-free, with Raman spectra collected at multiple points across the surface confirming a homogeneous monolayer. After annealing in air, two key features are observed: (i) numerous triangular openings are randomly distributed across the flake surface, and (ii) uniform etching occurs from the flake edges. All triangular pits are oriented opposite to the original growth direction of the flake. Closer inspection of the annealed flake reveals many small particles within the triangular pits (Fig. S1). The contrast in SEM images suggests that these particles consist of heavier elements, likely tungsten or tungsten oxide. What appear as “smooth edges” in Fig. 6c are, in fact, sharp triangular indentations, clearly visible in Fig. S1b and S2. Both the triangular pits and edge indentations display 60° angles, consistent with the hexagonal symmetry of the WS_2_ lattice.

The etching process of TMDCs by annealing in oxygen containing atmosphere is well studied and can be described by following chemical reaction:

1 WS_2_ + 2O_2_ → WO_*x*_ + 2SO_2_

CVD grown WS_2_ and other TMDCs may have edges terminated with transition metal (M) or with chalcogenide atoms (X). Three primary edge types exist in TMDCs: zigzag edges terminated by metal atoms (M-ZZ), zigzag edges terminated by chalcogen atoms (X-ZZ), and armchair (AC) edges.^29–31^ Because armchair edges preserve hexagonal symmetry, triangular pits cannot originate from AC terminations, allowing us to exclude AC edges as the source of the observed triangle openings. Zhou *et al.* reported similar behaviour for monolayer MoS_2_ annealed in oxygen, where aligned triangular pits formed above 350 °C along specific lattice planes.^32^ First-principles calculations indicated that transition metal oxidation is more favourable than chalcogen oxidation, resulting in pits aligned with the M-ZZ edges. During oxygen annealing, Mo is first oxidized to MoO_3_, followed by the formation of volatile SO_2_. For CVD-grown WS_2_, we observe inversely oriented triangular structures at the flake edges (Fig. 6), suggesting two distinct etching mechanisms. Triangular pits in the interior of the flake likely originate from oxidation at sulphur vacancies, whereas edge etching follows the chalcogen-terminated zigzag (S-ZZ) structure. This difference is presumably due to the nature of defects: edges predominantly contain dangling bonds that weaken S–W bonding, promoting sulphur oxidation, while interior pits initiate at sulphur-deficient sites.

### 3.2 Photoluminescence properties and defect formation

**Fig. 7:**
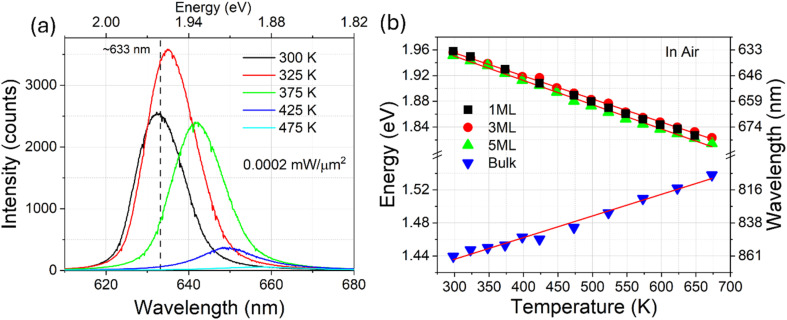
(a) Temperature dependent PL spectra taken from monolayer thick WS_2_ in air. The laser power density had to be constantly increased to obtain sufficient intensity at even higher temperatures and (b) the change of the peak position of the main emissions as a function of temperature in air. The dashed line in (a) indicates the room temperature direct band gap transition.

The impact of annealing in argon and air on the optical properties of WS_2_ was investigated using photoluminescence (PL) spectroscopy. In monolayer (ML) and few-layer WS_2_, the PL emission is dominated by radiative recombination of neutral (X^0^) and charged (X^+^/^−^) excitons, whereas bulk WS_2_ exhibits weak PL at ∼860 nm at room temperature, corresponding to indirect band-gap transitions (Fig. S3).^39^Fig. 7a shows high-temperature PL spectra of monolayer WS_2_ annealed in air. As expected, the PL intensity decreases and the emission peak redshifts with increasing temperature; similar behaviour was observed for 3 ML and 5 ML flakes. Due to the intensity decrease at elevated temperatures, the excitation laser power was increased for measurements taken above 475 K to maintain sufficient signal.

Fig. 7b summarizes the PL peak shift as a function of annealing temperature. The slope is consistent across all thin flakes, following the Varshni law.^40^ Samples annealed in argon exhibit significantly higher thermal stability, although PL spectra above 800 K could not be recorded due to substrate thermoluminescence (Fig. S4), which fully overlaps the trion emission. No new PL peaks associated with oxidation or defect formation were observed. Notably, excitation of WS_2_ in argon required an order-of-magnitude lower laser power to achieve similar intensity, indicating that oxygen strongly degrades the optical properties of the monolayer.

**Fig. 8:**
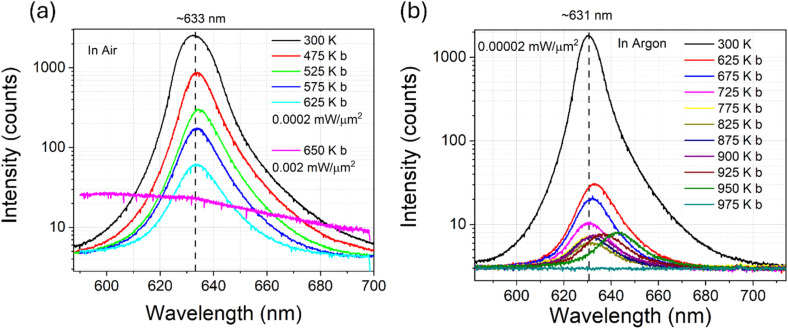
Room temperature PL taken from monolayer thick WS_2_ sample after each high temperature annealing step in air (a) and in argon (b). The *y*-axis is in log scale to account for the fast quenching of PL intensity with higher annealing temperature.

An initial increase in PL intensity at ∼325 K in air (Fig. 7a), absent in argon (Fig. S4a), suggests partial passivation of vacancy-related deep trap states by oxygen, leading to temporary enhancement of the emission. For bulk WS_2_ (Fig. S6), increasing temperature broadens the PL and induces new optical transitions at ∼750 nm and ∼840 nm. Unlike the direct-gap PL of few-layer flakes, the ∼840 nm peak exhibits a blue shift with temperature (Fig. 7b). This anomalous behaviour is reminiscent of observations in lead chalcogenides (PbS, PbSe, PbTe) and bulk black phosphorus and is attributed to the combined effects of thermal expansion, strong electron–phonon coupling, and large lattice anharmonicity.^41^ Instead of collecting PL at elevated temperatures, we recorded room-temperature PL after each annealing step. Fig. 8 shows the RT PL spectra of monolayer WS_2_ annealed in argon, compared with air-annealed samples. Because the RT PL after annealing below 600 K is nearly identical in both atmospheres, only spectra corresponding to higher-temperature annealing are shown in Fig. 8b. In air, the monolayer flake was already degraded after annealing at 650 K, whereas in argon the flake remained stable, allowing measurements up to higher temperatures. Annealing in argon up to 900 K resulted in a gradual decrease of PL intensity, but the peak position and spectral shape remained unchanged. The first significant redshift of the PL peak was observed after annealing at 925 K, with a more pronounced shift after 950 K. No luminescence was detected after annealing at 975 K due to complete flake degradation. These results further confirm that the thermal stability and preservation of optical properties in monolayer WS_2_ are strongly dependent on the annealing atmosphere. Annealing near the threshold degradation temperature leads to the formation of triangular pits, as illustrated in Fig. 6, with defect concentration increasing with both annealing time and temperature. Dangling bonds at these defect sites are known to trap electrons, and the PL emission from the flake edges is primarily associated with radiative recombination of charged trions.^42^ After annealing at 950 K, the PL peak is observed at 643 nm, consistent with the expected emission from negatively charged trions in monolayer WS_2_. Based on the presented data, the thermal stability of TMDCs strongly depends on the annealing conditions. The sample in argon can be annealed up to 950 K, while annealing in an oxidising atmosphere causes the triangular pit formation at 675 K. The earlier degradation at 650 K in air during PL can be attributed to the strain caused by constantly heating up and cooling down the sample, whereas in Raman, the temperature monotonically increased and the triangle pits were observed after annealing at 675 K.

To verify the role of defects in the oxidation of TMDC flakes, we investigated the influence of defect concentration on the thermal stability of WS_2_. Mechanically exfoliated monolayer and bilayer WS_2_ flakes were implanted with P ions at fluences of 5 × 10^12^ and 1 × 10^13^ cm^−2^, respectively. The sample implanted with the lower fluence remained stable up to 550 K, whereas samples implanted with 1 × 10^13^ cm^−2^ were fully oxidized above 500 K. These results demonstrate that ion-implanted samples exhibit significantly reduced thermal stability compared to non-implanted flakes. Ion implantation induces atomic displacements and vacancy formation, and in mono- and bilayer WS_2_ such defects act as catalytic centres for oxidation, promoting triangular pit formation at lower temperatures.

**Fig. 9:**
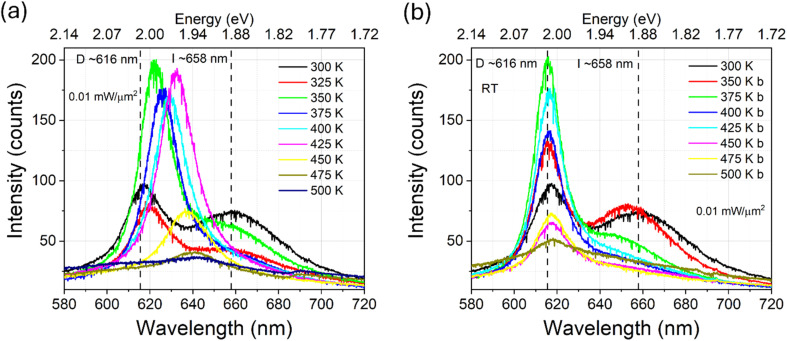
High temperature (a) and RT PL spectra (b) taken from mechanically exfoliated bilayer WS_2_ implanted with P-ions to a fluence of 1 × 10^13^ cm^−2^ after annealing in air.

Fig. 9 presents high-temperature and room-temperature PL spectra (indicated with b) obtained from P-implanted bilayer WS_2_. Phosphorus substituting at the sulphur site acts as an acceptor in WS_2_, and changes in PL emission provide insight into its electrical activation. The room-temperature PL spectrum exhibits two emission bands: one at ∼616 nm, attributed to the direct band-gap transition, and another at ∼658 nm, associated with the indirect band-gap transition.^34^ As shown in Fig. 9a, above 400 K only the direct band-gap emission remains visible. Due to the small energy separation between the indirect and direct valleys in bilayer WS_2_, increasing temperature leads to thermal redistribution of carriers, enhancing radiative recombination at the direct band gap while suppressing the indirect transition. A similar effect has been reported in indirect band-gap semiconductors with small valley separations, such as germanium and bulk MoSe_2_.^43,44^ Above 500 K, no PL signal is detected due to complete flake oxidation.

Room-temperature PL spectra recorded after each annealing step reveal that the direct band-gap emission initially increases, most likely due to partial annealing of ion-induced defects. Above 425 K, however, the PL intensity begins to decrease. In contrast, the emission associated with the indirect band gap gradually weakens and disappears completely after annealing above 400 K. The evolution of the RT PL spectra after annealing can be attributed to strain effects, doping variations, or a combination of both. The formation of triangular pits introduces edge states that can trap electrons, thereby modifying the average carrier concentration in the bilayer. Similar to germanium, bilayer WS_2_ can transition toward a pseudo-direct band-gap semiconductor when the electron concentration becomes sufficiently high.^45^ Thus, defect-induced carrier redistribution and edge-related charge trapping likely play a central role in the observed modification of the optical response.

## 4. Conclusions

We have systematically investigated the thermal degradation of WS_2_ under argon and air atmospheres, revealing a strong dependence of both thermal stability and lattice response on the annealing environment. Annealing in air not only significantly lowers the degradation threshold but also induces anisotropic thermal expansion in monolayer and few-layer WS_2_, attributed to oxygen interaction with the flake surface. In contrast, samples annealed in argon exhibit enhanced thermal robustness and nearly isotropic expansion behaviour.

Morphological analysis shows that triangular pits formed in the interior of the flakes are aligned along metal-terminated zigzag edges, whereas edge oxidation proceeds preferentially along S-terminated zigzag directions, indicating distinct defect-mediated etching mechanisms. Furthermore, ion-induced defects dramatically reduce thermal stability, confirming that vacancies and dangling bonds act as catalytic centres for oxidation.

These findings demonstrate that defect density and annealing atmosphere critically determine the structural and optical stability of WS_2_. Our results highlight the necessity of controlling defect formation and protecting flake edges—*e.g.*, *via* encapsulation—when designing 2D-material-based optoelectronic devices. Thermal processing steps, such as contact activation, must therefore be carefully optimized to preserve the intrinsic properties of TMDC layers.

## Author contributions

D. J., S. Z., and S. P. conceived the experiments and wrote the manuscript with input from all authors. P. W. provided the pristine WS_2_ samples, Y. L. provided the P-implanted WS_2_ samples, and U. K. performed the P-implantation. D. J. performed room-temperature and high-temperature Raman and PL characterization, and analyzed the results. D. J. captured the optical microscope images and O. S. captured the SEM images. S. Z. and S. P. arranged and revised the manuscript.

## Conflicts of interest

There are no conflicts of interest to declare.

## Supplementary Material

RA-016-D6RA02325H-s001

## Data Availability

The authors confirm that the data supporting the findings of this study are available within the article and its supplementary information (SI). Supplementary information is available. See DOI: https://doi.org/10.1039/d6ra02325h.
