# Supplementary material for: Thermal stability and anisotropic thermal expansion of WS2 annealed in different atmospheres
Source: RSC Adv. 2026 Jul 15;16(37):40505–15. doi: 10.1039/d6ra02325h (PMC13370266; doi:10.1039/d6ra02325h)
Supplement: Supplementary file 1 [file RA-016-D6RA02325H-s001.pdf]

Supplementary Information (SI) for RSC Advances.
This journal is © The Royal Society of Chemistry 2026

Supplementary Information

Thermal Stability and Anisotropic Thermal Expansion of WS_2 Annealed in Different Atmospheres

Doyeon Jin, Peiting Wen, Yi Li, Ulrich Kentsch, Oliver Steuer, Shengqiang Zhou, Slawomir Prucnal

1 Institute of Ion Beam Physics and Materials Research, Helmholtz-Zentrum Dresden-Rossendorf, 01328 Dresden, Germany

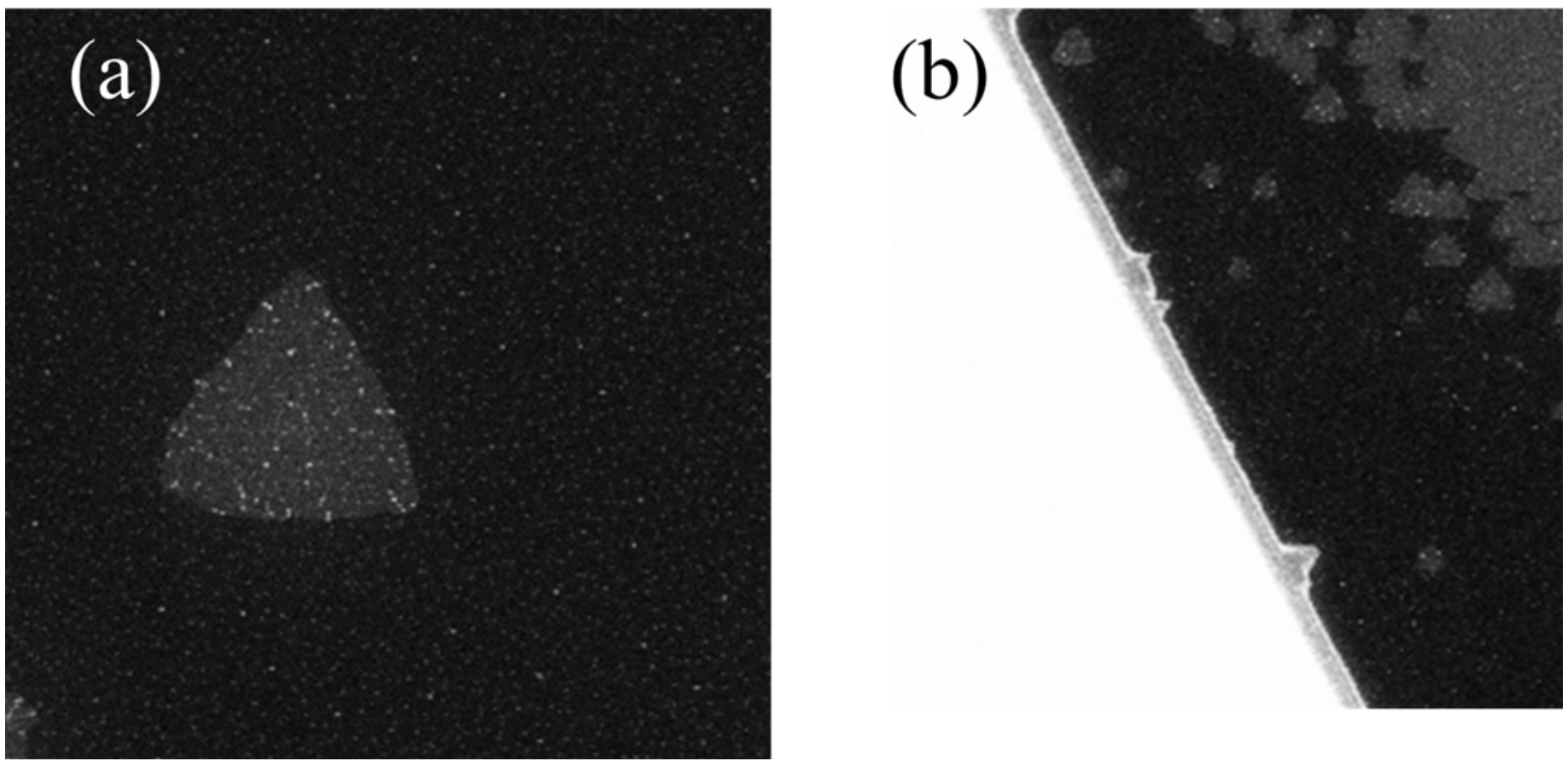

Figure S1. SEM images of triangle opening formed in monolayer thick WS_2 upon annealing in air at 400°C and the edge of the flake.

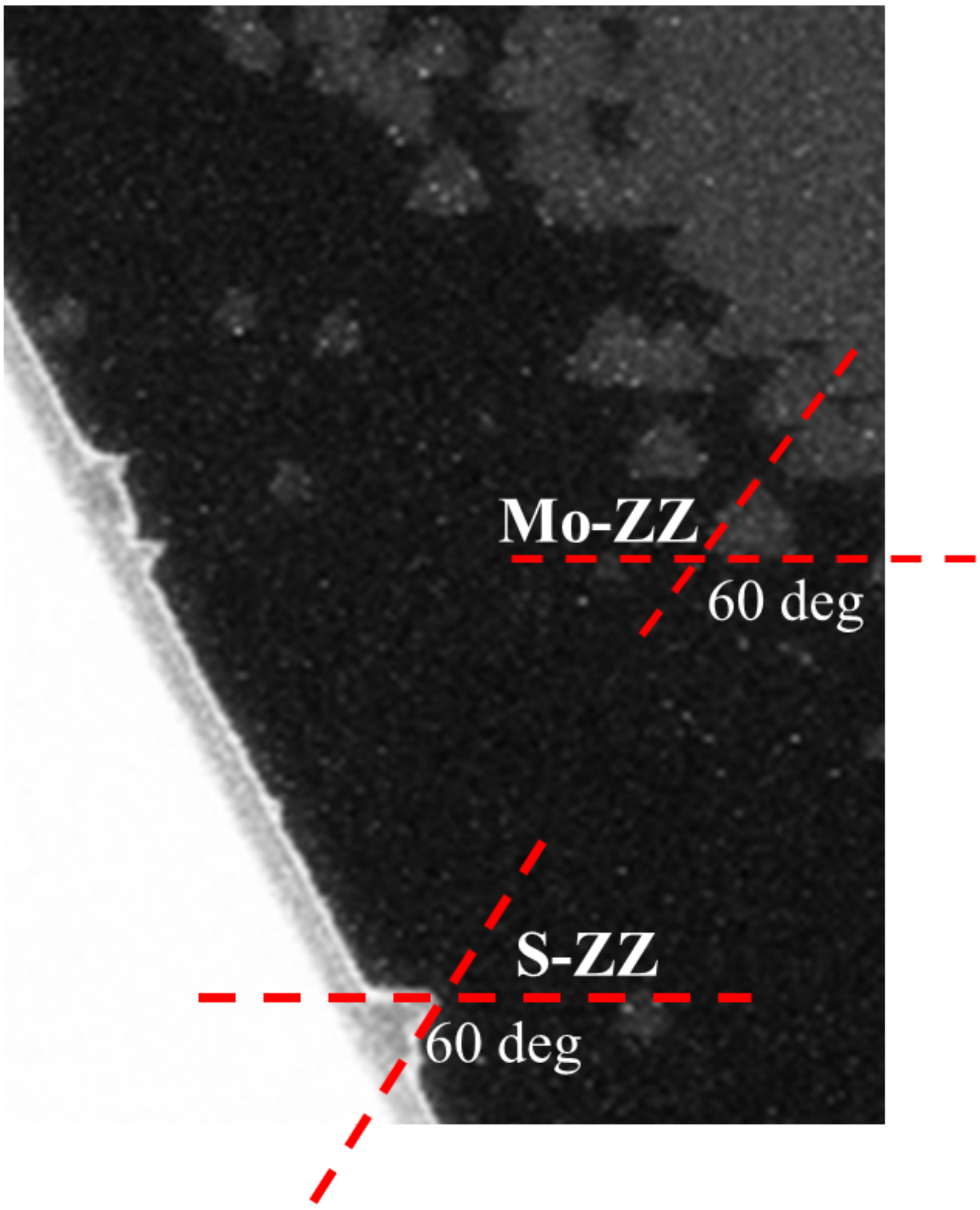

Figure S2. Schematic illustration of the etching mechanism of monolayer thick WS_2 in oxygen ambient at high temperature. The triangle holes follow the zigzag structure terminated by W atoms while the edges are etched by S-oxidation first and follow the S-termianted zigzag structure.

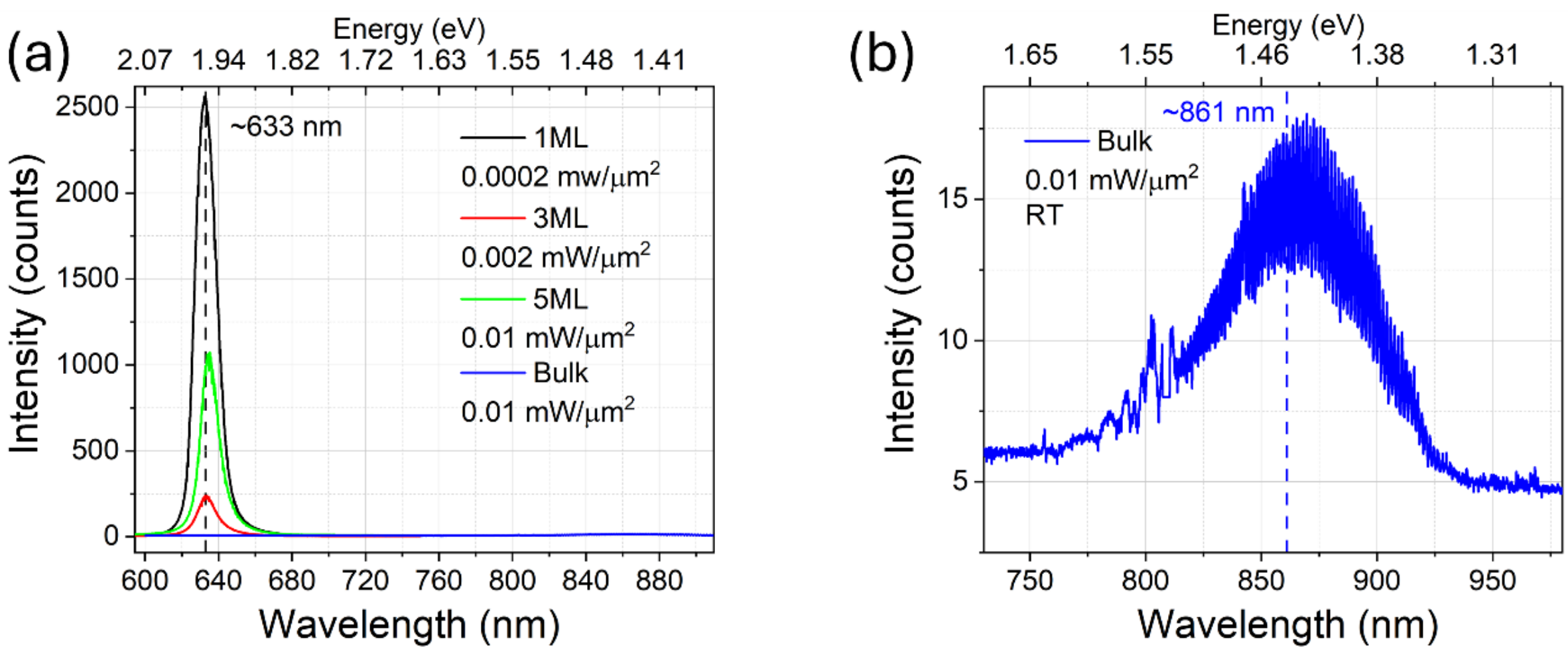

Figure S3. (a) Room temperature photoluminescence obtained from WS_2 with different thicknesses and (b) indirect band gap emission from bulk WS_2. The main PL emissions presented in (a) are due to trion emission at direct band gap.

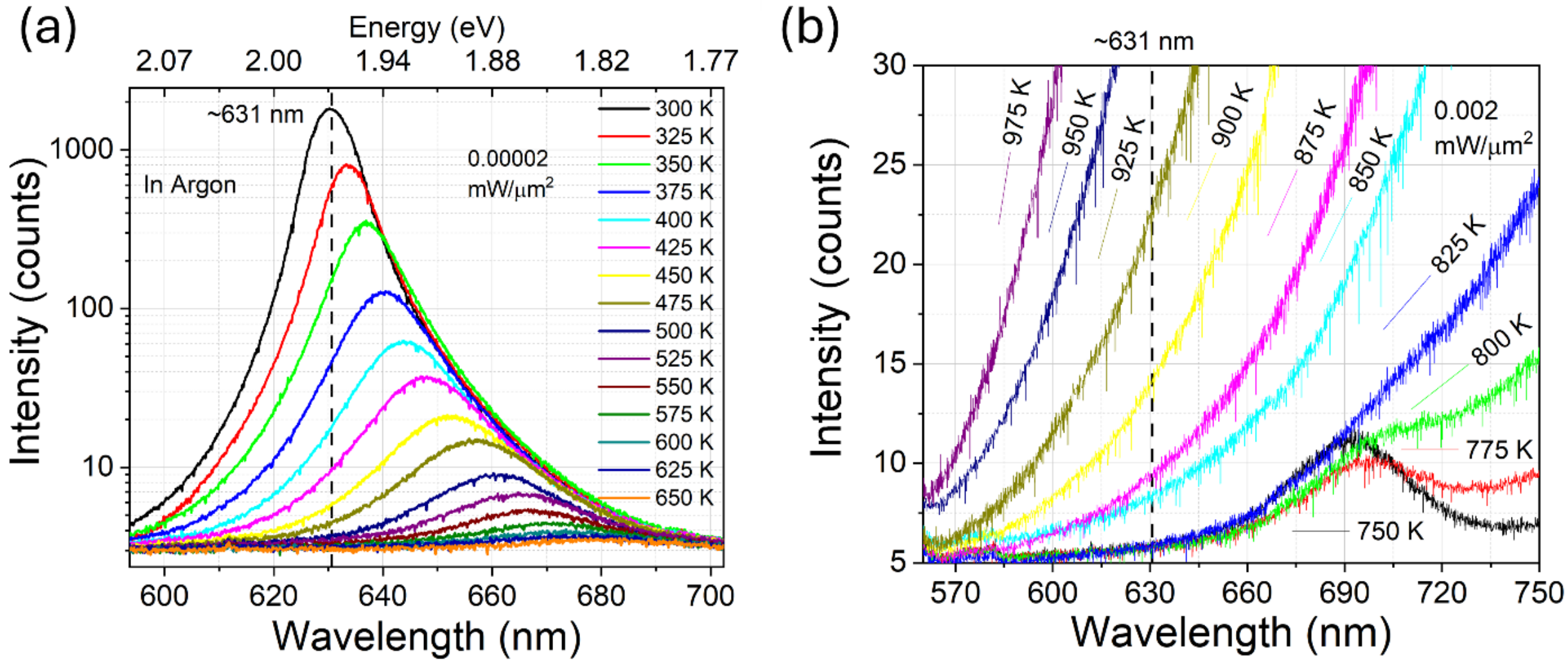

Figure S4. High temperature PL spectra taken from 1ML WS_2 in argon, which was still stable at 650 K (a). The y-axis is in log scale. However, above 800 K the PL emission is dominated by thermoluminescence from the substrate (b). The dashed black line around 631 nm is the room temperature PL peak position.

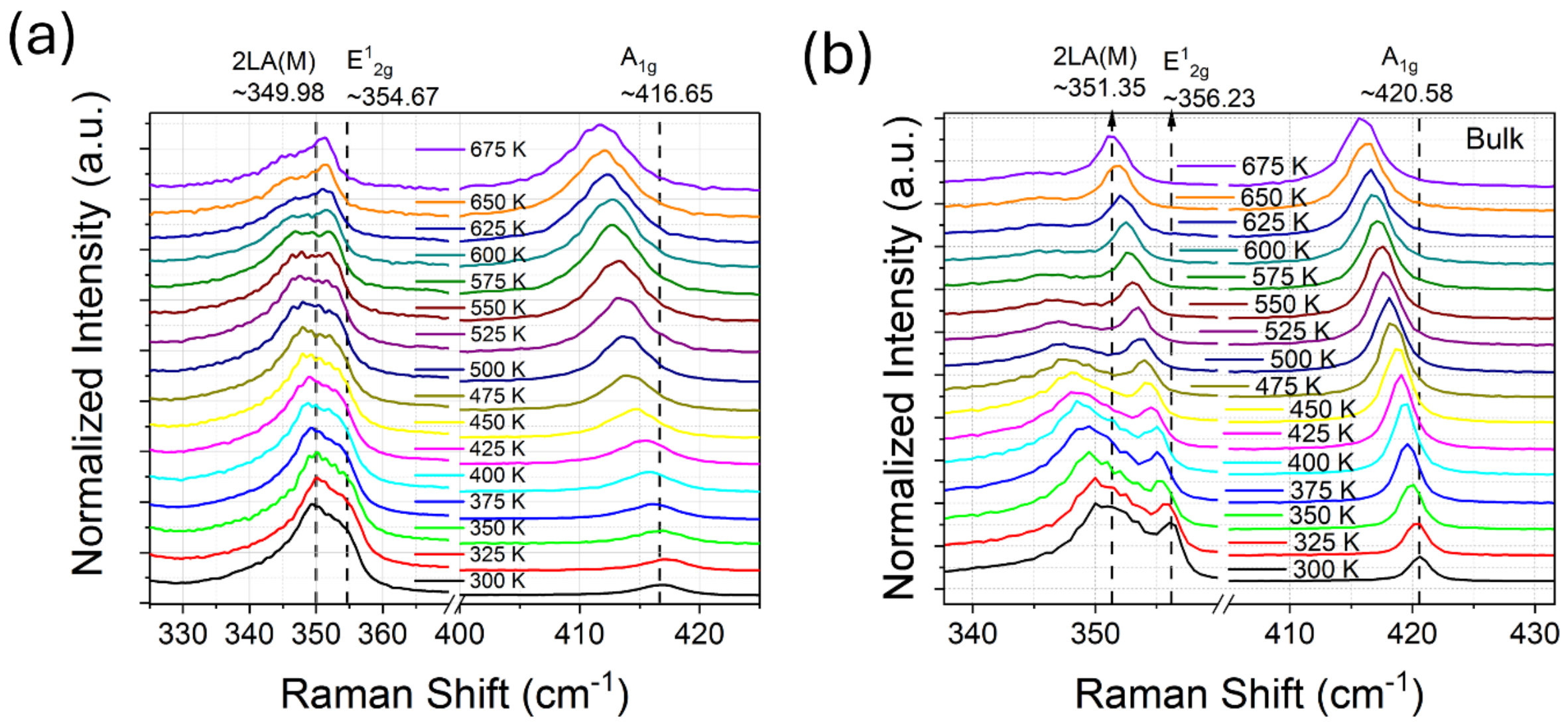

Figure S5. (a) Temperature dependent micro-Raman spectra taken in air ambient from monolayer thick WS_2 and from (b) bulk crystal. The spacing between 2LA(M) and E^1_{2g} phonon modes are noticeably larger in bulk.

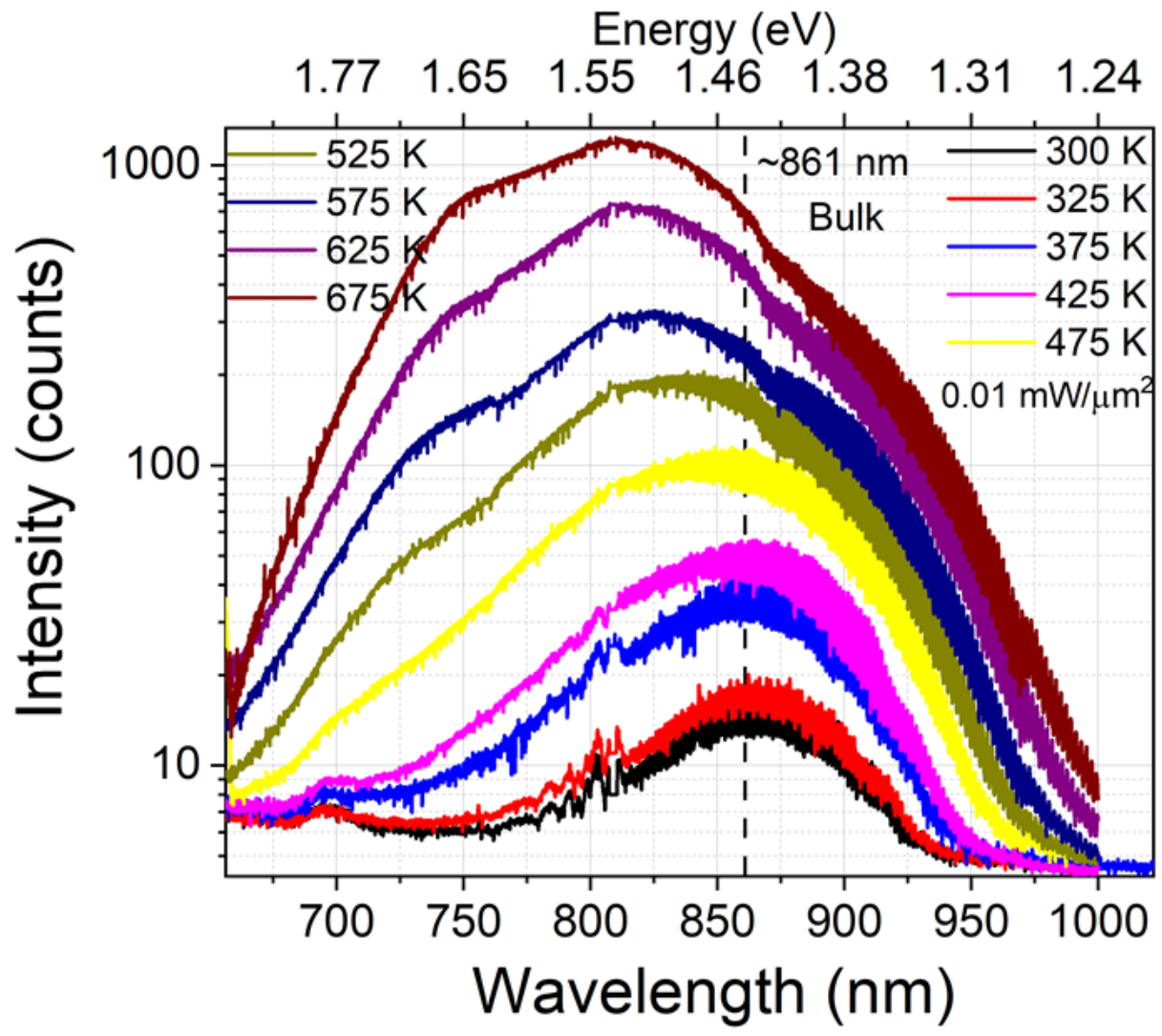

Figure S6. Temperature dependent PL spectra of bulk WS_2 crystal in air. The dashed black line is the PL peak position at RT. The intensity in the y-axis is in log scale for comparison.
